# All-Inside Posterior Cruciate Ligament Reconstruction Using Autologous Quadriceps Tendon–Bone Block

**DOI:** 10.1016/j.eats.2023.07.031

**Published:** 2023-10-30

**Authors:** Francesco Pettinari, Piero Franco, Lucia Conoscenti, Zyad Ayman Taha, Roberto Civinini, Fabrizio Matassi

**Affiliations:** Department of General Orthopedic, University of Florence, A.O.U. Careggi CTO, Florence, Italy

## Abstract

Posterior cruciate ligament ruptures account for nearly 20% of knee ligament lesions. These may be either isolated or occur as part of multiligament injuries. In most of the cases, conservative treatment is recommended, but when operative treatment is required, this is technically demanding. Several posterior cruciate ligament reconstructive techniques have been described, but some concerns still remain regarding graft choice, tunnels position, visualization of the posterior compartment and graft fixation. We describe an arthroscopic all-inside technique using a single-bundle autologous quadriceps tendon with patellar bone block.

The posterior cruciate ligament (PCL) is composed of 2 main bundles: anterolateral and posteromedial. The native ligament is extremely stiff, averaging 38 mm in length and 13 mm in diameter, and it is the primary restraint to posterior tibial translation.

Posterior cruciate ligament reconstruction (PCLR) procedures are challenging surgeries. Numerous reconstructive techniques have been described to treat PCL deficiency, including both single- and double-bundle reconstructions using open inlay or arthroscopic techniques.

Some evidence suggests that the use of autograft may reduce posterior laxity compared with allograft tendon; however, one of the main concerns is to obtain a thickness good enough to replace the native PCL.^1^^,^^2^

In this paper, we describe an anatomic, single-bundle, all-inside approach for arthroscopic PCLR using autologous quadriceps tendon–bone graft.

## Surgical Technique (With Video Illustration)

### General Preparation

After regional anesthesia, the patient is placed in the supine position; antibiotic prophylaxis is administered. A tourniquet is applied on the operative thigh and the knee is checked for full range of motion and confirmed to be stable on the foot roll at 90° of flexion.

### Surgical Approach

**Fig 1:**
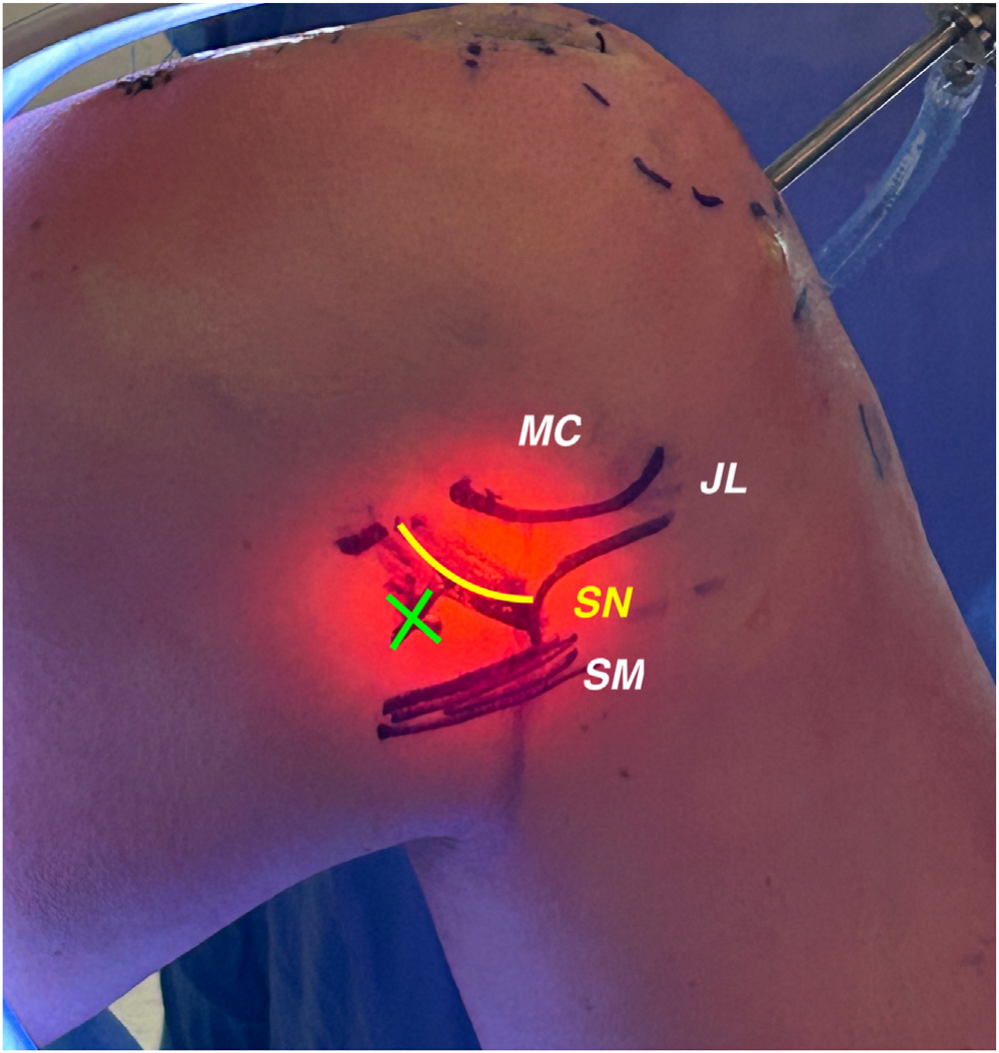
On external medial point of view the posteromedial arthroscopic portal (PM, green cross) is shown in the preoperative setting, in a left knee, flexed at 90°. The SN (yellow line) can be visualized on translucency with the help of an arthroscope. (JL, joint line; MC, medial condyle; SM, semimembranous tibial insertion; SN, saphenous nerve.)

A first, diagnostic arthroscopy is performed through standard anterolateral (AL) and anteromedial (AM) portals using a 30° arthroscope, in order to check for any associated intra-articular pathology. The femoral remnant of the anterolateral PCL bundle is dissected and the footprint is exposed, with care taken to maintain the meniscofemoral ligament untouched (Video 1). The posteromedial portal (PM) is then performed to clear the tibial PCL footprint, avoiding damaging the shiny white fibers of the posterior root of the medial meniscus (Fig 1).

### Graft Harvest

**Fig 2:**
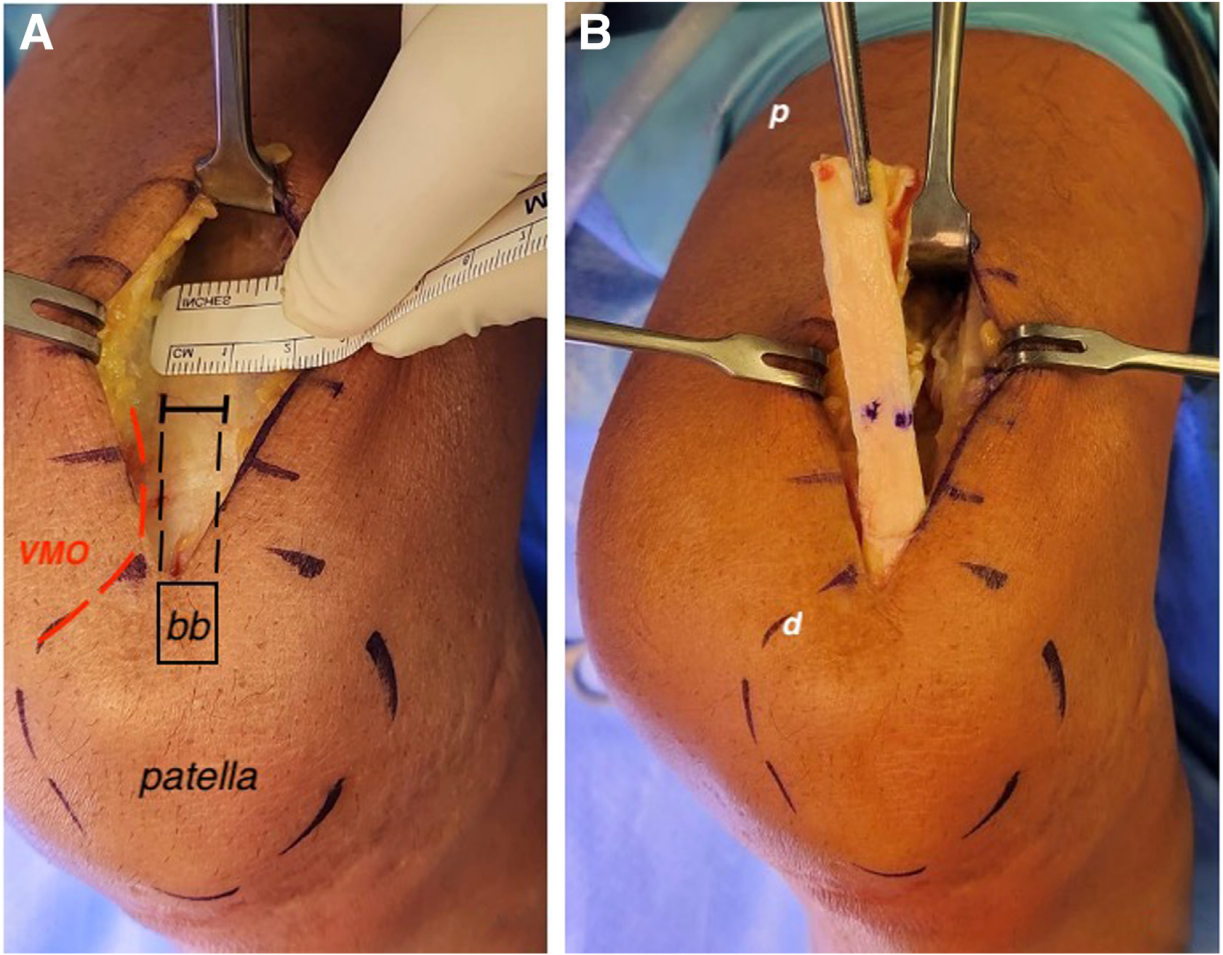
Quad tendon harvesting. The reader is looking at a left knee. A medial anterior 5-cm incision is made. (A) The tendon is incised slightly lateral to avoid the VMO fiber; the width is 10 mm. (B) The tendon is harvested in a proximodistal fashion, the bone–block (bb) is the last to be harvested; the “p” stands for the proximal and “d” stands for distal. (VMO, vastus medialis oblique.)

With the leg flexed at 90°, a longitudinal 5-cm incision is made from the edge of the proximal pole of the patella, at its midpoint extending proximally. Subcutaneous fat and paratenon directly over the quadriceps tendon (QT) are excised. The QT and vastus medialis oblique muscle are identified. Manipulation of the retractors and the use of the arthroscope under the subcutaneous tissue allow for adequate visualization of the proximal QT. We obtain a 10 mm-wide graft from the longest portion of the QT avoiding the VMO muscle fibers (Fig 2 and Table 1). A thin fat layer exists deep to the tendon and superficial to the capsule, which should alert the surgeon to avoid deeper dissection or the risk of capsular violation. The patella is marked and a 1-cm-wide and 2-cm-long bone block is harvested using an oscillating saw (Video 1).

**Table 1:** Pearls and Pitfalls of the Presented Technical note

Pearls	Pitfalls
Harvest QT in a proximal-distal fashion in order to cut proper size patellar bone block	Violation of the VMO must be avoided to minimize the risk of postoperative hematoma and quad weakness
Use the meniscofemoral ligament as a reference for PCL tibial tunnel preparation	Take care to obtain a graft length at least of 8 cm
Preserve PCL femoral remnant as a reference for femoral tunnel	Avoid damage to the shiny white fibers during PCL tibial footprint debridement, as they represent the medial meniscus root attachment
Introduce the graft from the AM portal to sit the bone block into the femoral tunnel and provide screw fixation from the AL portal	Tibial and femoral sockets must be long enough to permit the correct tensioning of the graft
Maintain the tibia at 90° of flexion in a reduced position during tibial fixation	To avoid the “killer turn,” use a metal trocar through the PM portal during the graft passage into the tibial socket to obtain a pulley effect

AL, anterolateral; AM, anteromedial; PCL, posterior cruciate ligament; PM, posteromedial; QT, quadriceps tendon; VMO, vastus medialis oblique.

Any capsular rents must be firmly closed and its integrity can be assessed by filling the joint with the arthroscopic fluid and examining for any leaks. The QT and its paratenon are sutured using absorbable wire and a sponge is placed under the incision delaying the subcutaneous and cutaneous suture at the end of the procedure.

### Graft Preparation

**Fig 3:**
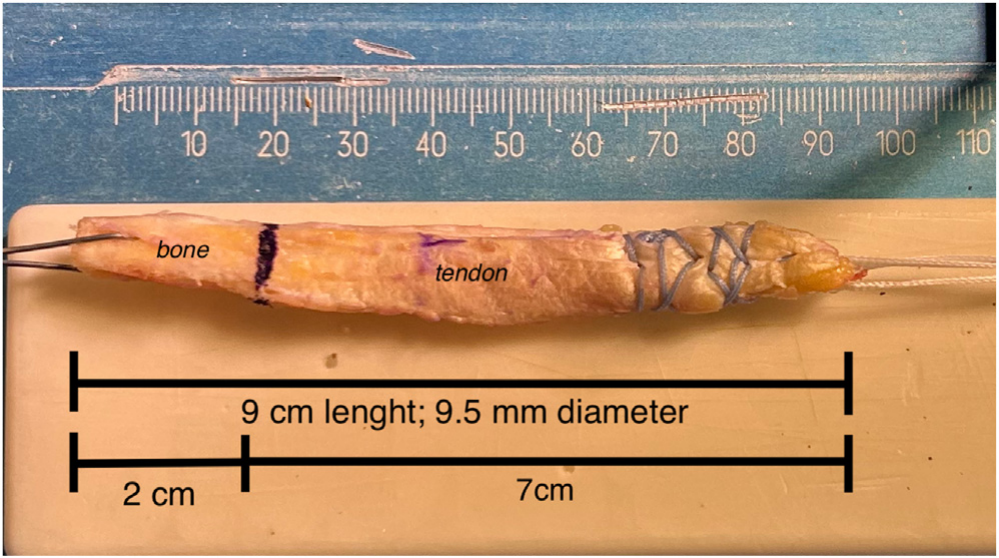
Quad tendon–patellar bone block harvested and its dimension: a 1.2-mm hole is performed at the center of the bone block, 0.8 mm metal wire carrier is passed through the hole. Bone–tendon border is marked with a pencil. The FiberTag Tightrope device (Arthrex, Naples, FL) is linked to the free tendon end of the graft.

The graft is prepared in the back table according to the GraftLink principles.^3^ The FiberTag Tightrope device (Arthrex, Naples, FL) is linked to the free end of the graft. A single hole is made at the center of the bone block using a 1.2-mm drill bit. An 0.8-mm metal wire is passed through the aforementioned hole, making a loop around the bone. The bone–tendon passage is marked with the marking pen and the graft diameter is then confirmed (Fig 3).

### Tunnel Preparation

**Fig 4:**
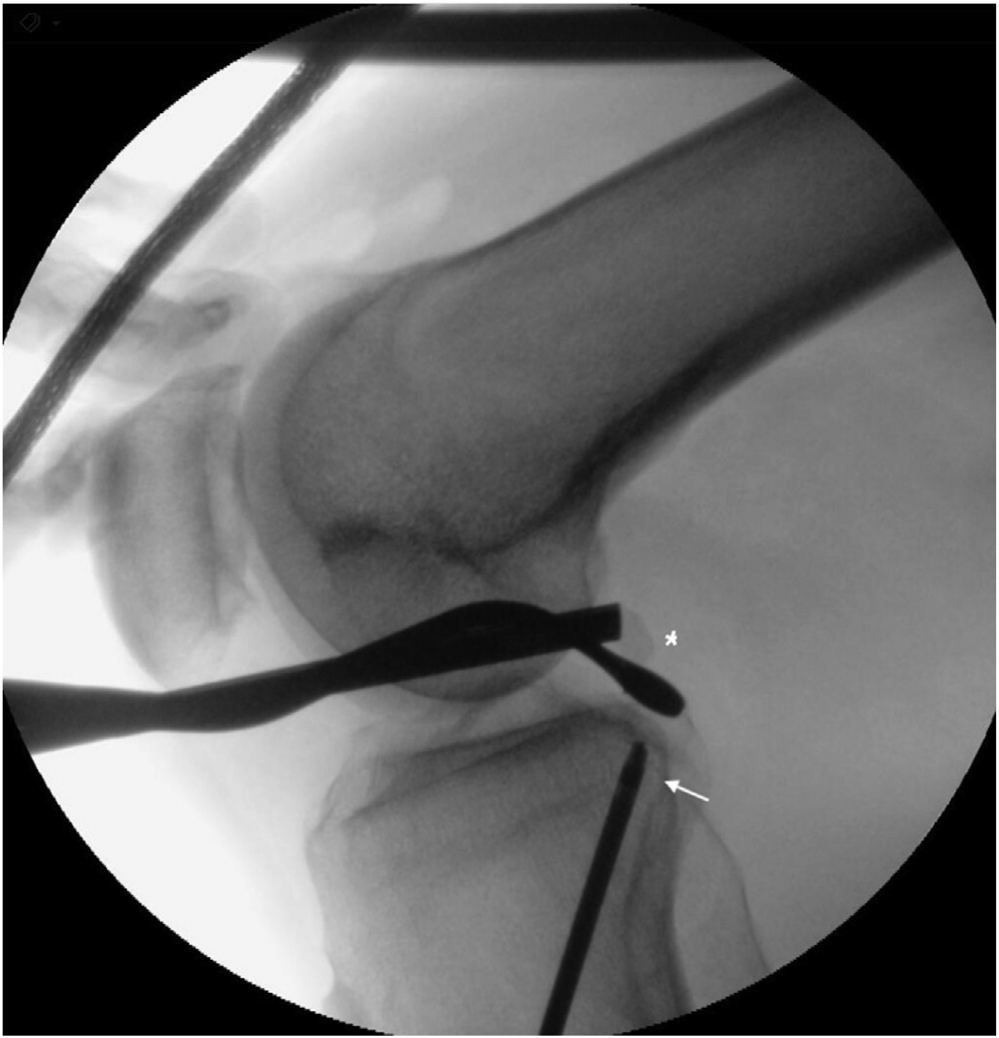
Intraoperative radiograph check for tibial PCL guide. It is highly recommended to ensure anatomical PCL insertion, and, to avoid potential vascular injuries, at this stage intraoperative radiograph should be mandatory. In the radiograph, the PCL tibial guide (white ∗) is aiming at the tibial PCL footprint, the FlipCutter (white arrow) is inserted through the tibia tunnel. (PCL, posterior cruciate ligament.)

The PCL tibial guide (Arthrex) is settled at 70° and it is inserted through the AM portal pointing to the tibial PCL footprint. A K-wire is introduced and proper position is checked under fluoroscopy control. The pin sleeve is then tapped into the tibia, the K-wire is removed and the 10-mm FlipCutter drill (Arthrex) is used to perform a 40 mm-long tibial socket (Fig 4). A suture shuttle is then passed into the tibial tunnel and retrieved from the PM portal (Video 1).

A 2.4-mm K-wire is pointed into the anterolateral PCL bundle through AL portal. For a better positioning, a cannulated drill is used as a guide. Then, a 30-mm femoral socket is drilled using progressive burrs until reaching a diameter 1 mm greater than the bone block. A suture shuttle is passed into the femoral tunnel and dragged through the PM portal. At this point, both femoral ant tibial suture shuttle are retrieved through the AM portal in order to avoid soft-tissue interposition between them.

### Graft Passage and Fixation

**Fig 5:**
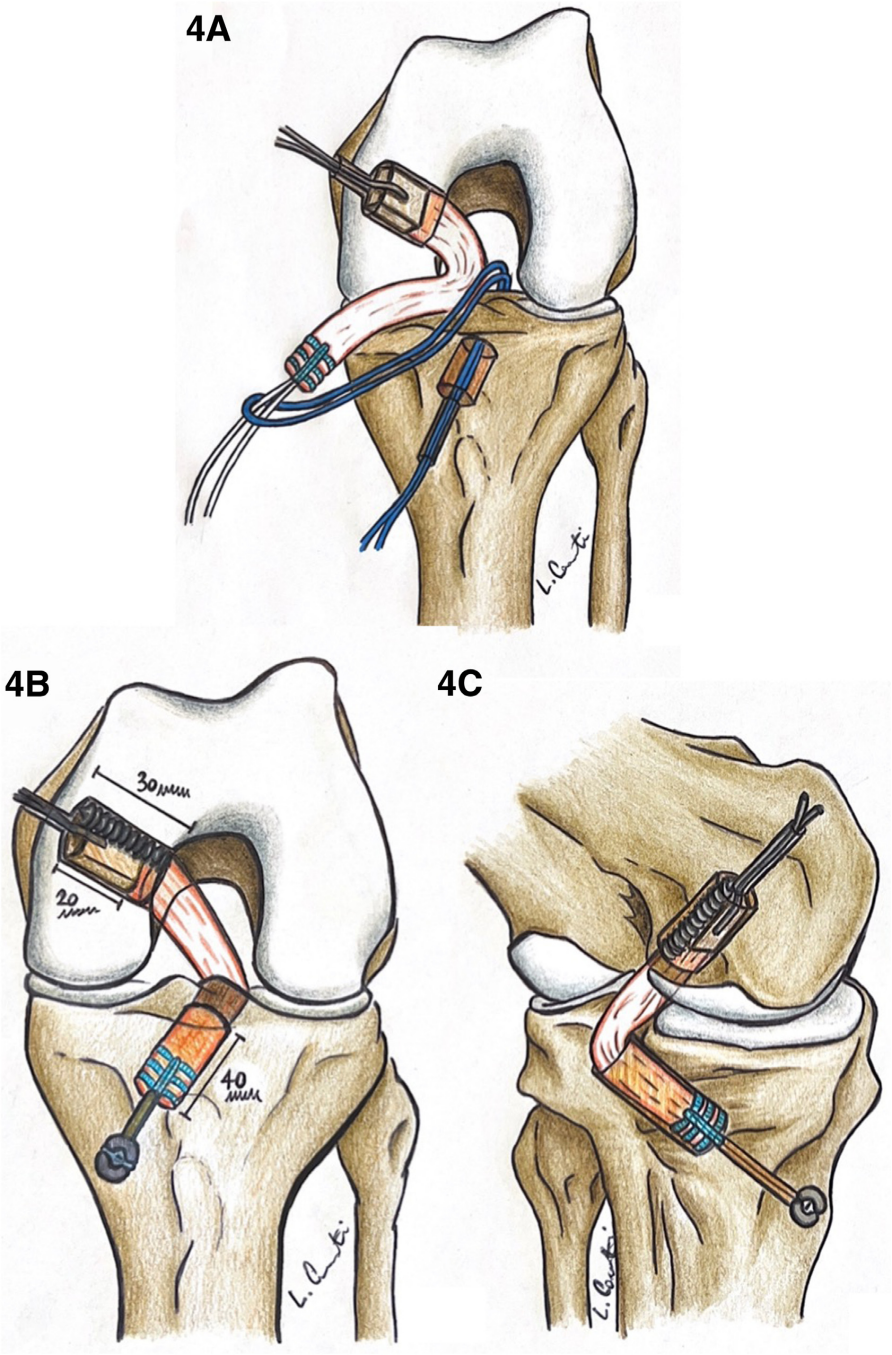
PCL graft fixation scheme. (A) Intermediate step of graft insertion: retrieved from the femur tunnel the graft is passed through AM portal it is fixed on the femur at its bony end with interference screw, the other extremity of the graft is still outside of the articulation and it lies though the AM portal. The graft is then retrieved from the tibial tunnel, during the passage the trocar is inserted through the PM portal and it is positioned on the posterior border of the tibial plateau to avoid any damage to the graft in this phase, the graft is fixed in the tibia with a suture button. (B) The final results in anterior view of QT graft fixation, with graft and tunnel measures. (C) Posteromedial view of final QT graft fixation. (AM, anteromedial; PCL, posterior cruciate ligament; PM, posteromedial; QT, quadriceps tendon.)

The femoral suture shuttle is pulled out as first in order to sit the patellar bone block into the femoral socket. The fixation is obtained by a 9×30 mm metal screw introduced through the AL portal. The screw is positioned against the posterior wall of the tunnel in order to push the graft anteriorly and restore the anatomical PCL insertion. Afterward, the tendinous extremity of the graft is pulled from the AM portal into the tibial socket. A metal trocar is introduced through the PM portal in order to obtain a pulley effect that minimize the “killer turn” effect (Video 1).^4^ The tibial fixation is reached by tightening the FiberTag device over a 14-mm round SutureButton (Arthrex). The knee is then moved through a cyclic range of motion multiple times and the tensioning of the FiberTag device is performed again, keeping the knee in a reduced position at 90° of flexion (Fig 5 and Table 2).

**Table 2:** Ten Key Points for a Correct Procedure

1. AM and AL portals to check for intra-articular injuries. Perform PM portal
2. Prepare PCL tibial and femoral footprints a. Femoral footprint: preserve meniscofemoral ligament b. Tibial footprint: preserve medial meniscal posterior root (look at the shiny white fibers)
3. Harvest 7-cm QT graft and 2-cm bony block, avoiding VMO muscle fibers
4. Arm FiberTag to the free end of the graft; metal wire through the bony block
5. Retrodrill 10-mm wide and 40-mm length tibial socket
6. Perform 11-mm wide and 25-mm length femoral socket through AL portal
7. Bone block introduced through AM portal into the femoral tunnel
8. Femoral fixation with interference screw through AL portal
9. Tibial graft passage through AM portal, cortical fixation tensioning FiberTag device and 14-mm suture button 10.Cycling, retensioning and final arthroscopic examination

AL, anterolateral; AM, anteromedial; PCL, posterior cruciate ligament; PM, posterolateral; QT, quadriceps tendon; VMO, vastus medialis oblique.

### End of the Procedure

**Fig 6:**
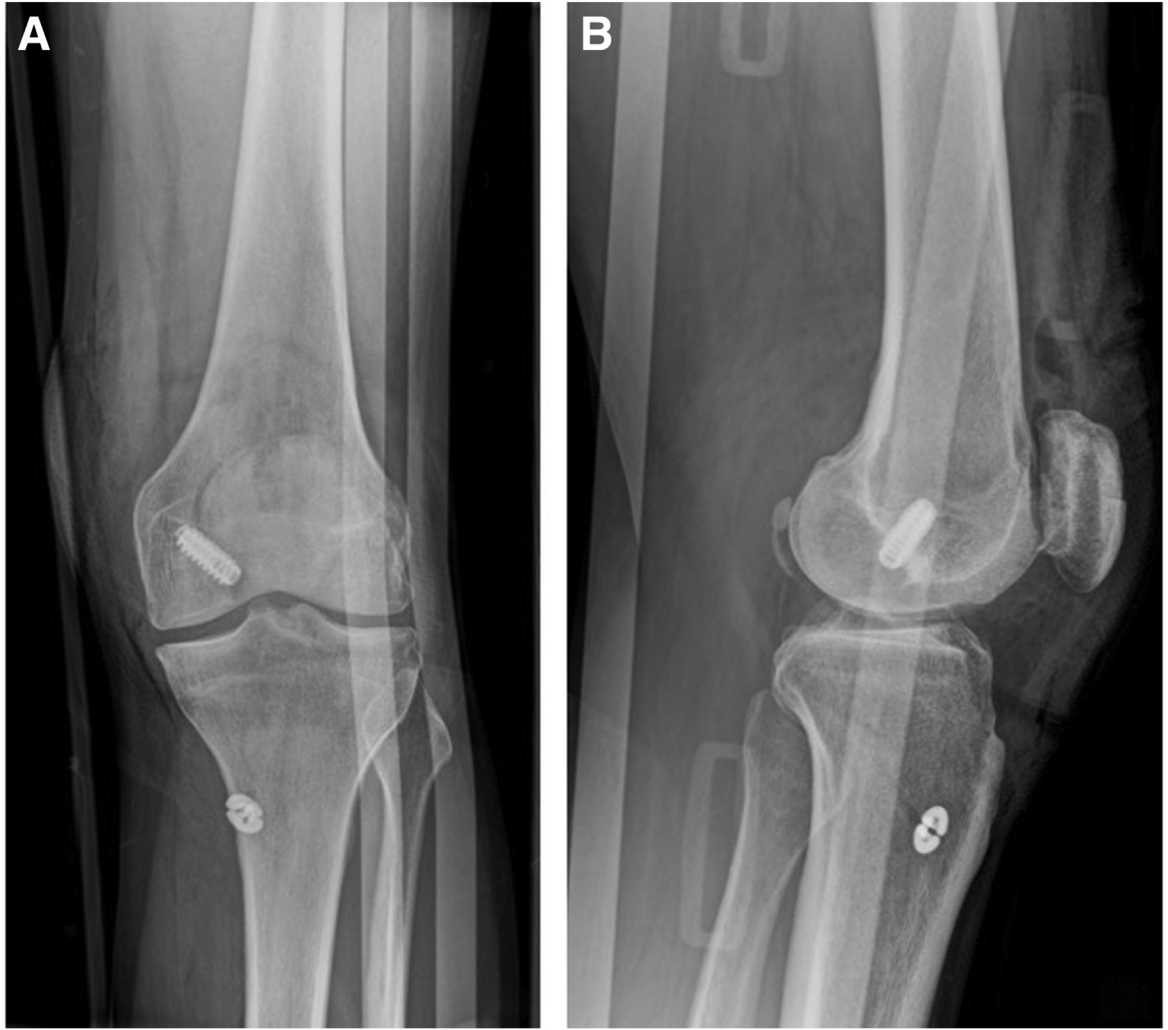
Postoperative radiograph; knee brace was already fitted. The radiograph shows no intra-articular exposition of the fixation device. (A) Anteroposterior view, (B) Lateral view.

At the end of the procedure, a last arthroscopic check is performed to ensure the correct tension of the graft. Torniquet is removed and meticulous hemostasis is performed. Wounds are irrigated and sutured. Elastic stocking is used in order to minimize knee and leg swelling. Final radiographs are performed (Fig 6).

### Postoperative Rehabilitation

A knee brace with proximal tibial support is applied for 30 days’ postoperatively. The patient is allowed to perform partial weight-bearing for the first month, and full weight-bearing is allowed after 30 days’ postoperatively. Quadriceps isometric exercise, straight-leg raising exercise, and passive range of motion should be initiated as early as possible.

## Discussion

PCLR is a technically demanding surgery, and many reconstructive techniques have been described. However, there are some concerns regarding the graft choice and the best surgical technique. No significant clinical differences have been shown between graft types used for PCLR, even though some evidence suggests that the use of autograft may result in reduced posterior laxity relative to allograft.^3^ The autogenous QT-B offers superior size and strength compared with other autografts, as the bone plug provides additional length and it allows accelerated bone-to-bone healing. The all-inside technique allows the creation of half tunnels both in the femur and in the tibia, preserving bone stock, minimizing the risk of tunnel widening and tunnel convergence in case of multiligamentous knee reconstruction. Moreover, the creation of sockets instead of full tunnels minimizes the “windshield effect,” and the all-inside technique allows the surgeon to reduce the “killer turn” encountered in the transtibial technique, which has been associated with graft failure (Table 3).^5^ Finally, the use of interference screws in the bony end of the graft lower the risk of tunnel enlargement as a stiffer fixation system has been used.^6^ The presented technique offers the advantages of bone–bone healing combined with an all-inside technique. However, the technique we described is not without limitations: compared with allograft, a proper graft diameter and length may be difficult to obtain with autogenous grafts (Table 3).^2^ Also, harvesting the QT-B could cause donor-site morbidity, risk of patellar fractures, and increased operative time (Table 1).

**Table 3:** Advantages and Disadvantages of the Presented Technical Note

Advantages	Disadvantages
Bone stock preservation	Technically demanding
Enhanced bone-to-bone healing	Increased operative time
Proper autograft diameter and length with bone block	Graft harvesting site local complications (patellar fractures, quad weakness)
Avoidance of killer turn during graft fixation in bone socket	Possible lateral condyle fracture during femoral tunnel preparation and screw insertion
The bone socket minimized the windshield effect of the graft	

## Supplementary Data

ICMJE author disclosure forms

Video 1 Surgical technique is described step-by-step, comprehensive of arthroscopic and external videos of the procedures. The presented cases is a 44-year-old male patient who suffers of knee instability after left knee sprain trauma occurred 1 year ago after traffic accident. Physical therapy was firstly addressed but failed to provide any improvement. Physical examination showed a slight knee effusion, complete range of motion (ROM), negative Lachman and anterior drawer tests, posterior drawer test +++, symmetrical dial test at 30° and 90°, negative valgus-varus stresses, and negative meniscal tests. MRI showed a grade 3 posterior cruciate ligament rupture.
